# Exploratory Analysis of the Role of Radiomic Features in the Differentiation of Oncocytoma and Chromophobe RCC in the Nephrographic CT Phase

**DOI:** 10.3390/life13101950

**Published:** 2023-09-23

**Authors:** María Aymerich, Alejandra García-Baizán, Paolo Niccolò Franco, Milagros Otero-García

**Affiliations:** 1 Diagnostic Imaging Research Group, Galicia Sur Health Research Institute, Hospital Álvaro Cunqueiro, 36312 Vigo, Spain; alejandra.garcia.baizan@sergas.es (A.G.-B.); milagros.otero.garcia@sergas.es (M.O.-G.); 2 Radiology Department, Hospital Álvaro Cunqueiro, 36312 Vigo, Spain; 3 Department of Diagnostic Radiology, IRCCS San Gerardo dei Tintori, Via Pergolesi 33, 20900 Monza, Italy; p.franco@campus.unimib.it

**Keywords:** diagnostic radiology, computed tomography, artificial intelligence, texture analysis, radiomics, renal cell carcinoma, oncocytoma

## Abstract

In diagnostic imaging, distinguishing chromophobe renal cell carcinomas (chRCCs) from renal oncocytomas (ROs) is challenging, since they both present similar radiological characteristics. Radiomics has the potential to help in the differentiation between chRCCs and ROs by extracting quantitative imaging. This is a preliminary study of the role of radiomic features in the differentiation of chRCCs and ROs using machine learning models. In this retrospective work, 38 subjects were involved: 19 diagnosed with chRCCs and 19 with ROs. The CT nephrographic contrast phase was selected in each case. Three-dimensional segmentations of the lesions were performed and the radiomic features were extracted. To assess the reliability of the features, the intraclass correlation coefficient was calculated from the segmentations performed by three radiologists with different degrees of expertise. The selection of features was based on the criteria of excellent intraclass correlation coefficient (ICC), high correlation, and statistical significance. Three machine learning models were elaborated: support vector machine (SVM), random forest (RF), and logistic regression (LR). From 105 extracted features, 41 presented an excellent ICC and 6 were not highly correlated with each other. Only two features showed significant differences according to histological type and machine learning models were developed with them. LR was the better model, in particular, with an 83% precision.

## 1. Introduction

Renal cell carcinoma (RCC) is the most common type of kidney cancer in adults, accounting for approximately 90% of all cases [1]. RCC typically affects individuals between the ages of 50 and 70 years and is more prevalent in men than women [2]. The etiology of RCC is not clear; however, certain risk factors have been identified, such as smoking, obesity, or hypertension [3]. RCC exhibits different degrees of malignancy and benignity based on its subtypes. The most prevalent subtype is clear cell RCC (ccRCC), which constitutes about 70–80% of all RCC cases and has a malignant biological behavior. Other less common subtypes include papillary RCC, which can be further divided into types 1 and 2 according to histologic and morphologic characteristics, or chromophobe RCC (chRCC). Additionally, some renal tumors exhibit benign characteristics, such as renal oncocytomas (ROs) or angiomyolipomas [4].

One of the challenging aspects of a renal cell carcinoma is that it often goes undetected in its early stages, as symptoms may be mild or absent. The diagnosis of RCCs involves imaging examinations, such as ultrasound (US), computed tomography (CT) scans, or magnetic resonance imaging (MRI), and in most cases it is incidentally detected in a study for another pathology [5].

Among renal tumors, chRCCs account for approximately 6–8% of all renal tumors, while ROs constitute 3–7% of them [6]. Both tumors originate from intercalated cells in the collecting duct and share morphologic, histologic, and immunohistochemical features. Despite this, ROs are considered benign tumors, while chRCCs have malignant biological behavior. In fact, although being frequently indolent and having a more favorable prognosis compared to other RCC subtypes, chRCCs have the potential for metastatic spread. However, at diagnosis, chRCCs are usually low-stage cancers, and lymph node and distant metastases are not frequent.

With the increasing use of cross-sectional abdominal imaging, chRCCs are often detected as incidental findings, since they are commonly asymptomatic. Nevertheless, they can sporadically manifest, with clinical symptoms including abdominal or flank pain, abdominal mass, hematuria, and systemic symptoms, such as fever, fatigue, cachexia, and weight loss [7]. Similarly, ROs are usually diagnosed incidentally and patients do not usually complain of urologic symptoms. When symptomatic, ROs may manifest as gross hematuria, flank pain, frequent urinary tract infections, and a palpable abdominal mass upon a physical examination [8].

Routine clinical imaging techniques, including US, CT, and MRI, cannot completely distinguish malignant chRCCs from benign ROs, since they also share many similar radiological characteristics, such as well-defined borders, homogeneous enhancement, and low vascularity on a contrast-enhanced CT [9]. On a CT, chRCCs typically appear as well-circumscribed lesions with smooth margins and a heterogeneous appearance. Calcifications are not frequent, being seen in one-third of cases. A central scar is present in 19–34% of cases, with a spoke-wheel pattern of enhancement observed in a minority of cases. On non-contrast CT scans, chRCCs are iso-attenuating or slightly hyperattenuating compared with the surrounding renal parenchyma. On contrast-enhanced CT images, chRCCs show a moderate enhancement, typically less than ccRCCs [10,11]. Typical RO radiological features include well-circumscribed contours, a homogeneous appearance, and a central stellate scar, with an absence of necrosis and hemorrhage [12].

Several investigations aimed to detect radiological features for differentiating ROs from RCCs, particularly based on CT imaging. Notably, the presence of a central scar is considered a characteristic feature for an RO; however, it is not pathognomonic. In a histologic analysis, it was reported that a fibrous scar was present in 45% of cases of RO; however, it was also present in 23% of chRCC cases [13]. Moreover, many RCC subtypes can exhibit areas of necrosis at imaging, which mimics the presence of a scar, representing an additional limit for the utility of this feature for reliable CO diagnoses.

Therefore, the preoperative differentiation between chRCCs and ROs cannot be performed accurately solely based on the imaging findings [14]. Renal mass biopsy is considered the most reliable diagnostic modality but can be complicated by the histopathological similarities between ROs and some variants of chRCCs. For these reasons, both tumors are traditionally treated with radical nephrectomy. Nevertheless, an accurate preoperative diagnosis of ROs may lead to avoiding unnecessary nephrectomies, preferring nephron-sparing approaches—such as partial nephrectomy, cryotherapy, or radiofrequency—or even active surveillance [15,16].

Given these challenges, a multidisciplinary approach is often necessary for an accurate diagnosis. Personalized medicine can play a significant role in overcoming the challenges of differentiating between chRCCs and oncocytomas by tailoring diagnostics to individual patients. In this context, radiomics is a rapidly evolving field in diagnostic imaging that creates computational techniques to extract and analyze a large number of quantitative features from medical images [17,18,19]. Through radiomic approaches, specific imaging biomarkers associated with each tumor type can be identified and applied to the classification of lesions. In the field of solid renal masses, some works have analyzed the usefulness of radiomics in the classification of RCCs or the staging of ccRCCs [20,21,22,23,24].

Radiomics has the potential to significantly impact the differentiation between chRCCs and ROs, as well as other cancer types by extracting quantitative imaging features from standard medical imaging studies and selecting imaging biomarkers that may help in distinguishing between the two tumor types, enhancing the diagnostic accuracy [25].

While radiomics holds great promise, it is still a developing field and its full potential in differentiating chRCCs from oncocytomas is yet to be fully realized. As with any advanced medical technology, thorough validations and standardizations are essential to ensure that radiomic features are reliable and clinically applicable. This is a preliminary study for the evaluation of the role of radiomic features in the differentiation between chRCCs and ROs using machine learning models.

## 2. Materials and Methods

### 2.1. Patients

**Figure 1:**
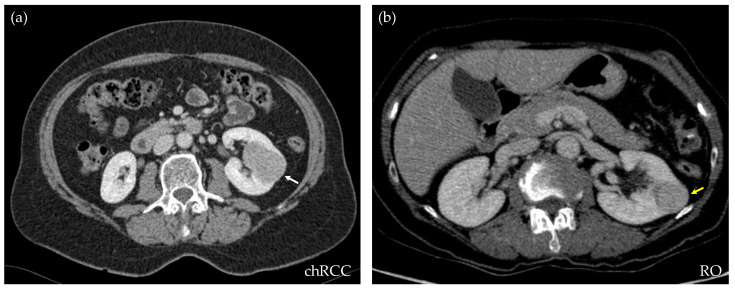
CT axial sections of the nephrographic phase of two subjects, both showing a well-defined, homogeneous, and hypodense renal lesion. After nephrectomy, the histopathologic results demonstrate that the lesion on the left ((**a**), *white arrow*) is a chromophobe RCC, while the lesion on the right ((**b**), *yellow arrow*) is a renal oncocytoma. Through diagnostic imaging, the criteria cannot be established to differentiate the benign from the malignant mass.

For this retrospective work, 173 CT studies of solid renal masses were collected at our center, from 2016 to 2021. All of them had histological confirmations. Excluded cases were those that did not have a complete image study or the confirmation of the histological type of the tumor. From the whole database, 109 were clear cell RCCs, 17 papillary type I, 9 papillary type II, 19 chRCCs, and 19 ROs. For the purpose of this study, only chRCCs and ROs were selected. This observational study received the approval of the Local Ethics Committee under the code 2021/008. Table 1 shows the demographic data corresponding to the included patients and lesions. Figure 1 shows an axial section of the nephrographic series of an RO and a chRCC, illustrating the difficulty that exists for their differentiation through diagnostic imaging.

### 2.2. Image Acquisition

The CT imaging study corresponding to the time point at which the solid renal mass was first detected, which generally occurred incidentally, was selected. The nephrographic contrast phase (90 s after intravenous contrast) was selected in each case. Since the cases were obtained from the clinical routine of our center, the studies were acquired with different CT scanners, including a Somaton X.cite (Siemens Healthcare, Forchheim, Germany), a Somaton Drive (Siemens Healthcare, Forchheim, Germany), a LightSpeed VCT (GE Healthcare, Milwaukee, WI, USA), and a Philips Ingenuity (Philips Healthcare, Eindhoven, the Netherlands).

### 2.3. Image Analysis

The nephrographic phase of each study was anonymized and uploaded to the Quibim Precision 2.8 platform (Quibim S.L., Valencia, Spain), with CE marking and IBSI compliant, where lesions were delimited and the radiomic features were extracted. Radiomic features were grouped in shape, first order, gray-level co-occurrence matrix (GLCM), gray-level run-length matrix (GLRLM), gray-level size-zone matrix (GLSZM), gray-level dependence matrix (GLDM), and neighboring gray-tone difference matrix (NGTDM). The full list is presented in Supplementary Materials. To assess the reliability of the radiomic features, the intraclass correlation coefficient (ICC) for each feature was calculated from the segmentation done by three different radiologists with different degrees of expertise (resident, junior, and senior). They performed a volumetric segmentation of the lesions and the radiomic features were extracted using the aforementioned platform. For the feature extraction, the image intensity was normalized, and voxels were isotropically resampled to 1 × 1 × 1 mm^3^. The software internally removed the outliers and it set the distance to neighbor to 1.

### 2.4. Statistical Analysis and Feature Selection

In this work, R software (4.0.2 version) was used for the statistical analysis. ICCs among the three radiologists were calculated using the icc function from the irr package with model and type arguments as the two-way and agreement, respectively. Only features with an ICC ≥ 0.90, considered as excellent, were selected. The selection of radiomic variables for the elaboration of classificatory models was based on the criterion of an excellent ICC and, later, those highly correlated radiomic variables were eliminated, leaving only one feature for each group. Then, the statistical distribution of the features (parametric and non-parametric) was analyzed, and it was determined if there were significant differences between these variables and the histological type (chRCC vs. RO). Variables were presented as means and standard deviations (for Gaussian distributions) or medians and interquartile ranges (non-parametric distributions). To compare the means or medians in each case, a significance level of 0.05 was established and Student’s t-tests were performed for the variables with normal distributions and the Mann–Whitney U test for the non-parametric ones. Finally, only the features that met all the criteria for the ICC, non-correlation, and statistically significant difference according to the histological type were chosen. Different classification models were trained with the selected radiomic variables.

### 2.5. Model Elaboration

For the elaboration of different machine learning models, Python code (3.11.2 version) was employed. The data were split into training and test groups in an 80–20% proportion. The cross-validation parameter was set to 5. The data included in the database were transformed by subtracting the mean and normalizing this value to the unit variance. In particular, the following models were selected: support vector machine (SVM), random forest (RF), and logistic regression (LR). To optimize the hyperparameters of different models, a grid search space was defined, using the f1-score as the scoring parameter. The performance of each model was evaluated in the training dataset and the best performing hyperparameter combination was selected. The models were then applied to the test dataset and accuracy; f1-score, precision, and recall, among other parameters, were calculated using the classification report. The receiver operating characteristic (ROC) curves of the training and test datasets were also plotted for the three models and the area under the curve (AUC) with the 95% confidence interval of these representations was calculated. Supplementary Figure S1 depicts the scheme of the model elaboration and test.

## 3. Results

According to Table 1, which includes the demographic information of both subgroups, no statistically significant differences can be identified between the two groups. Therefore, they were considered comparable to each other.

**Figure 2:**
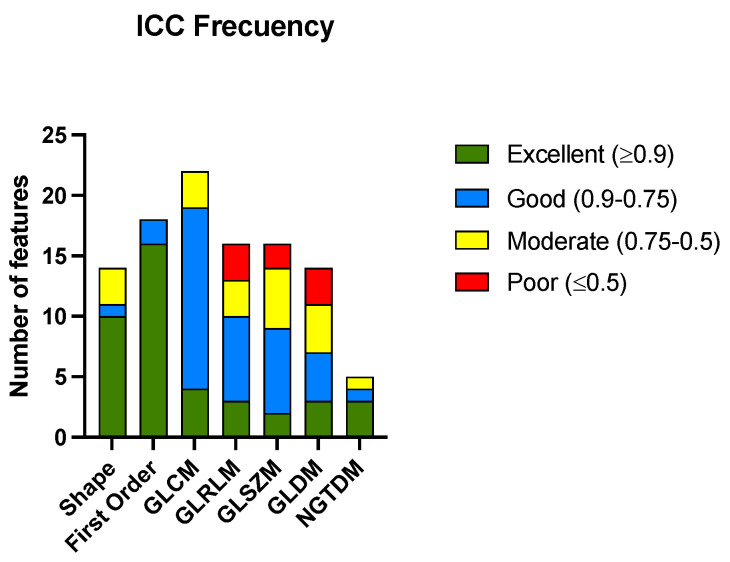
Histogram of the number of radiomic features with their corresponding ICC codes pooled for the groups of radiomic features. Gray-level co-occurrence matrix (GLCM), gray-level run-length matrix (GLRLM), gray-level size-zone matrix (GLSZM), gray-level dependence matrix (GLDM), and neighboring gray-tone difference matrix (NGTDM).

After calculating the ICC for all the radiomic features among the three radiologists, 41 features met the criteria for excellent ICCs. The ICC was considered excellent if it was equal to or greater than 0.9, good if it was between 0.90 and 0.75, moderate if it was between 0.75 and 0.5, and poor if it was equal to or less than 0.5. The complete list of the radiomic features analyzed is presented in Supplementary Materials. Figure 2 shows the histogram of the classification of the ICCs of the features according to the group to which they belong. Table 2 shows the numerical results of the analysis.

The features from the shape and first-order groups presented a higher percentage of excellent correlations between the different radiologists that performed segmentations. The group of features derived from the NGTDM matrix also had the most variables with excellent ICCs; although, it must be considered that the number of variables in this case was small. There were only three groups of features (GLRLM, GLSZM, and GLDM) that showed a percentage of variables with a poor correlation.

**Figure 3:**
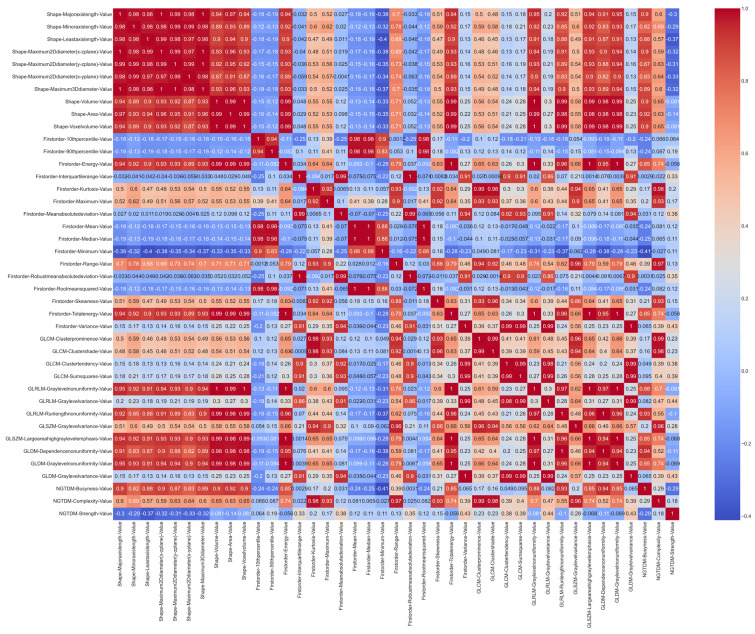
Correlation matrix based on the Pearson’s method with the features that meet an excellent ICC standard.

From the 41 radiomic variables that met an excellent ICC among the radiologists, the correlations that existed between them were analyzed. Figure 3 shows the correlation matrix using the Pearson’s method.

As depicted in Figure 3, the shape variables are highly correlated with each other. Once the Pearson’s correlation value was calculated, those groups of variables that were highly correlated were eliminated and only one representative feature of each cluster was selected. A value of 0.9 was established as the correlation threshold for this selection.

From the 41 variables with excellent ICCs, six radiomic variables were selected since they were not highly correlated with each other. In particular, one from the shape group, four from the first-order group, and one from the NGTDM group. Only one of the variables related to high-order features was selected. The type of distribution followed by these variables was determined using the Kolmogorov–Smirnov test. Only the 10th percentile (first order) and minimum (first order) presented normal distributions. Using the corresponding tests, we analyzed whether there were statistically significant differences between the two groups for each of the variables. The results are shown in Table 3.

**Figure 4:**
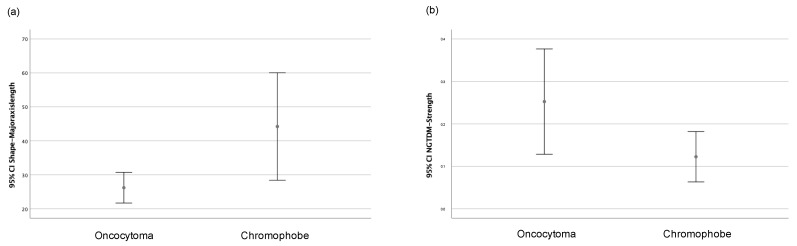
Graph of 95% confidence intervals for (**a**) major axis length (shape) and (**b**) strength (NGTDM) grouped according to the histological type (oncocytoma or chromophobe).

Only the major axis length (shape) and strength (NGTDM) features showed statistically significant differences depending on the histological type, whether chRCCs or ROs. Figure 4 shows the graphs of the 95% confidence intervals for both variables. Therefore, and considering the number of cases that were collected, these two radiomic variables were selected for elaborating the machine learning classificatory models.

**Figure 5:**
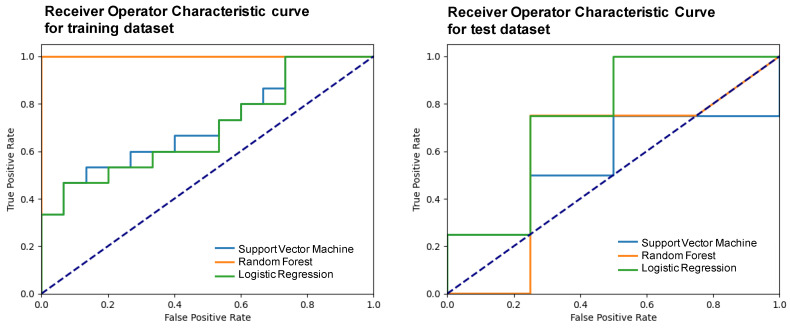
Receiver operator characteristic (ROC) curves of the different models for the training and test subsets.

The performance of the trained models was evaluated using the mean test score and its standard deviation, obtaining a score of 0.38 ± 0.15 for the support vector machine, 0.35 ± 0.23 for the random forest, and 0.53 ± 0.29 for the logistic regression. The three models were elaborated upon with the optimal hyperparameters and using a five-fold validation. Table 4 shows the metrics obtained after developing the machine learning models using the two selected variables and applying them to the test subset. Figure 5 presents the receiver operator characteristic (ROC) curve for the training and test subsets.

Based on the metrics summarized in Table 4 and on the number of cases available, it was considered that the LR was the best machine learning model that allowed classifying between chRCCs and ROs using only two variables: one associated with the size of the lesion and another purely radiomic. In particular, with this model, an 83% precision was obtained.

## 4. Discussion

In this work, we analyzed the role of radiomic features in the classification of ROs and chRCCs from the CT nephrographic series. Using the inter-observer correlation coefficient (ICC), the Pearson’s correlations of the variables, as well as their statistical significances according to the histologist group, two features were selected (major axis length and strength). With them, different machine learning models were trained, obtaining a precision of 83% in the case of a logistic regression model. This was a preliminary study whose main objective was to analyze the most promising radiomic features for the elaboration of further sophisticated classification models, with a higher number of cases from several centers that will eventually serve as support for clinical decisions in diagnoses by imaging.

Few studies solely focus on the radiomic differentiation between chRCCs and ROs, with high heterogeneity between them [25]. Alhussaini et al. [26] only used one axial cross-section of each scan using two cohorts, one in the nephrographic phase (NP) and the other in the corticomedullary phase (CMP), obtaining the lowest performance in the combined cohort and the best predictive performance for the nephrographic group. In this study, only the nephrographic phase was used for the segmentation of the lesion. Although the corticomedullary phase was also used in other studies, most renal masses were discovered incidentally in CT studies scheduled for another cause [8]. Therefore, the CMP phase was not available in most of them. In this work, a precision value of 83% was obtained using only two variables and a single CT phase, which can eventually make the results on the role of variables more transferable to clinical routines. There are also works that do not use the nephrographic phase and suggest its usefulness in the classification problem [27].

Li et al. [28] used the CMP, NP, and excretory (EP) phases for the differentiation between chRCCs and ROs, obtaining high sensitivity values. The central point of their work was the comparison of filtered and unfiltered radiomic variables, while our work used a single phase and focused on the role that these features played in the differentiation of these subtypes. Baghdadi et al. [29] used convolutional neural networks for this purpose, thus increasing the complexity of the model and making its explainability difficult by not analyzing the role of radiomic variables in decision making. The work of Deng et al. [30] obtained high specificity values; however, their sensitivity value was 34%. Xiao Li et al. [31] extracted the features of enhanced triphasic CT images, with good results; however, they only focused on the differential diagnosis of ROs and chRCCs in patients with a central scar.

The dimensionality reduction in the radiomic features through the ICC is a common procedure in radiomics workflow since it provides valuable information on the robustness of the variables within a manual segmentation and eliminates the features whose numerical values are significantly altered, depending on the physician that defines the lesion [32]. In this work, a volumetric segmentation of the whole renal mass was performed, in contrast to other works where only a segmentation of the largest slice was performed, and, thus, information for the lesion was neglected [26]. The selection of features that were not highly correlated was also necessary to avoid the redundancy of information.

In particular, in our study, major axis length (shape) and strength (NGTDM) were selected as final radiomics features. Although it is not clear, some studies suggest that the size of the chRCC is larger than that of the RO [33]. The strength feature belongs to the NGTDM group that quantifies the differences between the gray level of a voxel and the mean gray level of its neighboring voxels within a predefined distance, according to Amadasun and King [34]. Strength is defined as a measure of the primitives in an image. High strength values are related to defined and visible primitives, corresponding to a volume with a slow change in intensity but larger coarse differences in gray-level intensities. [35]. In some studies, the NGTDM group of variables is not considered for the radiomic analysis of renal tumors [36]; although, it seems that the features associated with this matrix may have classifying utility. To achieve a greater understanding of the biological mechanisms underlying the patterns captured by the radiomic features, some works combine radiomics with other types of data, such as genomics or proteomics [37]. This can help bridge the gap between radiomics and biological interpretations, providing a more comprehensive understanding of a disease processes; however, it is not the scope of this study.

Other works reflect that the first-order radiomic features (linked to the histogram of the VOI) are commonly used for the elaboration of classification models to distinguish between chRCCs and ROs [36,38]. In our case, they were not employed because they did not show a statistical significance according to the histopathological group; however, in previous steps, four first-order non-correlated features with excellent ICCs were selected. In the case of having a greater number of cases, the inclusion of these features in the elaboration of the machine learning model could be assessed. Regarding the employed models, random forest shows similar values to the logistic regression in terms of accuracy, f1-score, and precision. However, the percentage of model performance, as well as the AUC value, NPV, and precision show better results in the case of logistic regression; therefore, in this work it is presented as the most promising model in terms of classification. Other different classification models can provide better results if a larger number of cases are available. Furthermore, models where the selection of features is conducted during the training of the model instead of pre-selecting them can be developed; however, this increases the complexity of the model and makes the interpretation of this exploratory analysis difficult.

This study has several limitations. It is a single-center, retrospective study with a small number of cases; although, it must be taken into account that the number of RO cases per year and center are not usually very high, as it is not a frequent pathology. Ideally, a future model would have to be developed and validated with a larger number of cases. Nevertheless, the main objective of this work was a preliminary analysis of the role played by radiomic features in the classification of ROs vs. chRCCs. Furthermore, since the cases were acquired in a clinical routine, not all the CT studies were performed on the same CT scanner. Even though there have been several initiatives in recent years [39,40,41], the field of radiomics still does not have a clear standardization regarding the acquisition of images for different machines, among other aspects. Radiomics-based models offer a non-invasive approach to aid in the diagnosis and characterization of tumors, reducing the need for invasive procedures, such as biopsies, in some cases. Despite the promising potential, it is essential to acknowledge that machine learning models in medical imaging, including radiomics-based models, require rigorous validation and testing in diverse patient populations before clinical implementation.

By integrating radiomic classification models with traditional diagnostic and imaging methods, personalized medicine holds the promise of improving diagnostic accuracy, treatment outcomes, and overall patient care in the context of differentiating chromophobe RCCs from oncocytomas. In this work, a simple classificatory model was developed using the CT nephrographic phase to analyze the role of radiomic features and their ability to differentiate between those types of solid renal masses.

## Figures and Tables

**Table 1:** Demographics of the final database.

	Oncocytoma	Chromophobe
Number of cases	19	19
Median age (range)	69 (86–57)	70 (85–38)
Sex		
Female	11 (57.9%)	8 (42.1%)
Male	8 (42.1%)	11 (57.9%)
Laterality		
Right	8 (42.1%)	11 (57.9%)
Left	11 (57.9%)	8 (42.1%)

**Table 2:** Number of variables and percentage of radiomic variables within each group of features that meet the different ICC criteria. Data are displayed as numbers (percentages).

Radiomic Group	Excellent	Good	Moderate	Poor
Shape	10 (71.42)	1 (7.14)	3 (21.43)	0 (0)
First order	16 (88.89)	2 (11.11)	0(0)	0 (0)
GLCM	4 (18.18)	15 (68.18)	3 (13.64)	0 (0)
GLRLM	3 (18.75)	7 (43.75)	3 (18.75)	3 (18.75)
GLSZM	2 (12.50)	7 (43.75)	5 (31.25)	2 (12.50)
GLDM	3 (21.43)	4 (28.57)	4 (28.57)	3 (21.43)
NGTDM	3 (60.00)	1 (20.00)	1 (20.00)	0(0)

**Table 3:** Radiomic features that have an excellent ICC and are not highly correlated. Numerical values of the variables according to histological type are presented as means (standard deviations) or medians (interquartile ranges). Features that present statistically significant differences according to histological type are indicated with an *.

Radiomic Feature	Oncocytoma	Chromophobe	*p*-Value
Major axis length (shape)	25.62 (10.41)	32.09 (22.62)	0.02 *
10th percentile (first order)	734.28 (99.87)	732.86 (105.68)	0.96
Interquartile range (first order)	62.50 (20.48)	52.19 (21.77)	0.07
Kurtosis (first order)	3.47 (1.52)	3.65 (1.72)	0.28
Minimum (first order)	563.96 (108.25)	557.75 (149.95)	0.89
Strength (NGTDM)	0.15 (0.35)	0.06 (0.17)	0.04 *

**Table 4:** Classification report for the test dataset for each model. The main metrics are represented: accuracy, f1-score, precision, recall, specificity, negative predictive value (NPV), and area under the curve (AUC). Metrics are presented as mean (standard deviation).

Model	Accuracy	F1-Score	Precision	Recall	Specificity	NPV	AUC (95% CI)
Support vector machine	0.50 (0.10)	0.47 (0.12)	0.50 (0.23)	0.50 (0.10)	0.25 (0.20)	0.60 (0.38)	0.56 (0.21–0.70)
Random forest	0.75 (0.16)	0.75 (0.20)	0.75 (0.22)	0.75 (0.16)	0.63 (0.25)	0.54 (0.14)	0.59 (0.24–0.92)
Logistic regression	0.75 (0.21)	0.73 (0.21)	0.83 (0.16)	0.75 (0.19)	0.63 (0.23)	0.80 (0.20)	0.75 (0.55–1.00)

## Data Availability

The data presented in this study are available on request from the corresponding authors. The data are not publicly available due to the local Ethics Committee’s decision.

## Supplementary Materials

The following supporting information can be downloaded at: https://www.mdpi.com/article/10.3390/life13101950/s1, Figure S1: Diagram of the machine learning models development using 5 cross validation; Table S1: Complete list of the radiomic features that were extracted with Quibim Precision software, version 2.9.5.
