# Supplementary material for: Exploratory Analysis of the Role of Radiomic Features in the Differentiation of Oncocytoma and Chromophobe RCC in the Nephrographic CT Phase
Source: Life (Basel). 2023 Sep 23;13(10):1950. doi: 10.3390/life13101950 (PMC10607929; doi:10.3390/life13101950)
Supplement: Supplementary file 1 [file life-13-01950-s001.zip › life-2609208-supplementary.pdf]

Supplementary Materials

I.a.:Complete list of radiomic features

Table S1: Complete list of the radiomic features that were extracted with Quibim Precision software.

List of radiomic features	
Shape	Elongation<br>Mayor axis length<br>Minor axis length<br>Sphericity<br>Flatness<br>Least axis length<br>Maximum 2D Diameter xy<br>Maximum 2D Diameter xz<br>Maximum 2D Diameter yz<br>Maximum 3D diameter<br>Volume<br>Area<br>Surface to volume ratio<br>Voxel volume
First Order	10th percentile
	90th percentile
	Energy
	Entropy
	Interquartile range
	Kurtosis
	Maximum
	Mean absolute deviation
	Mean
	Median
	Minimum
	Range
	Robust mean absolute deviation
	Root mean squared
	Skewness
	Total energy
	Uniformity
	Variance
Gray Level Cooccurrence Matrix (GLCM)	Autocorrelation
	Joint average
	Cluster prominence
	Cluster shade
	Cluster tendency
	Contrast
	Correlation
	Difference average
	Difference entropy

	Difference variance
	Joint energy
	Joint entropy
	Informational measure of correlation 1
	Informational measure of correlation 2
	Inverse difference moment
	Inverse difference moment normalized
	Inverse difference
	Inverse difference normalized
	Inverse variance
	Maximum probability
	Sum entropy
	Sum squares
Gray Level Run Length Matrix (GLRLM)	Gray level non uniformity
	Gray level non uniformity normalized
	Gray level variance
	High gray level run emphasis
	Long run emphasis
	Long run high gray level emphasis
	Long run low gray level emphasis
	Low gray level run emphasis
	Run entropy
	Run length nonuniformity
	Run length non uniformity normalized
	Run percentage
	Run variance
	Short run emphasis
	Short run high gray level emphasis
	Short run low gray level emphasis
Gray Level Size Zone Matrix (GLSZM)	Gray level non uniformity
	Gray level non uniformity normalized
	Gray level variance
	High gray level zone emphasis
	Large area emphasis
	Large area high gray level emphasis
	Large area low gray level emphasis
	Low gray level zone emphasis
	Size zone non uniformity
	Size zone non uniformity normalized
	Small area emphasis
	Small area high gray level emphasis
	Small area low gray level emphasis
	Zone entropy
	Zone percentage
	Zone variance

Gray Level Dependence Matrix (GLDM)	Dependence entropy
	Dependence non uniformity
	Dependence non uniformity normalized
	Dependence variance
	Gray level non uniformity
	Gray level variance
	High gray level emphasis
	Large dependence emphasis
	Large dependence high gray level emphasis
	Large dependence low gray level emphasis
	Low gray level emphasis
	Small dependence emphasis
	Small dependence high gray level emphasis
	Small dependence low gray level emphasis
Neighboring Gray Tone Difference Matrix (NGTDM)	Busyness
	Coarseness
	Complexity
	Contrast
	Strength

I.b: Formula for the calculation of the ICC

$$ICC\ (2{,}1) = \frac{MSBS - MSE}{MSBS + (k-1)MSE + (\frac{k}{n})(MSBS - MSE)}$$

Being MSBS mean square between features, MSE mean square error, k number of raters and n number of features. From: doi: 10.1371/journal.pone.0219854

I.c: Scheme of the machine learning model elaboration and testing

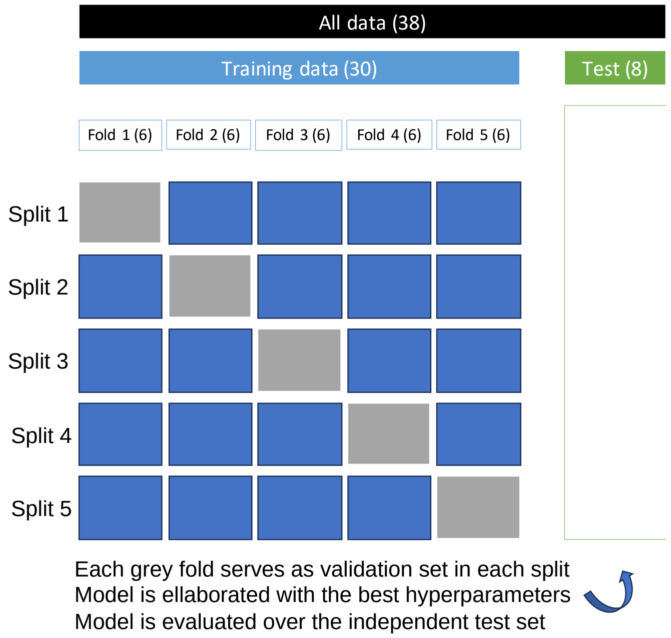

Figure S1.: Diagram of the machine learning models development using 5 cross validation. The whole dataset is divided in training and test. Once the model with the best hyperparameters is elaborated, it is testes over the test dataset.
